# Production of putative enhanced oral cholera vaccine strains that express toxin-coregulated pilus

**DOI:** 10.1371/journal.pone.0175170

**Published:** 2017-04-06

**Authors:** Caitlyn A. Hauke, Ronald K. Taylor

**Affiliations:** Department of Microbiology and Immunology, Geisel School of Medicine at Dartmouth, Hanover, New Hampshire, United States of America; Instituto Butantan, BRAZIL

## Abstract

The use of whole cell killed (WCK) oral cholera vaccines is an important strategy for cholera prevention in endemic areas. To overcome current vaccine limitations, we engineered strains of *V*. *cholerae* to be non-toxigenic and to express the protective protein colonization factor, toxin-coregulated pilus (TCP), under scale-up conditions potentially amenable to vaccine production. Two *V*. *cholerae* clinical strains were selected and their cholera toxin genes deleted. The *tcp* operon was placed under control of a rhamnose-inducible promoter. Production and stability of TCP were assessed under various conditions. The strains lack detectable cholera toxin production. The addition of 0.1% rhamnose to the growth medium induced robust production of TCP and TcpA antigen. The strains produced intact TCP in larger growth volumes (1 L), and pili appeared stable during heat-killing or acid treatment of the bacterial cultures. To date, no WCK cholera vaccines have included TCP. We have constructed putative strains of *V*. *cholerae* for use in a vaccine that produce high levels of stable TCP antigen, which has not previously been achieved.

## Introduction

*Vibrio cholerae* is a globally important pathogen, causing an estimated 2.8 million cases of cholera and approximately 91,000 deaths in endemic countries, with an additional 87,000 cases and 2,500 deaths in non-endemic countries [1]. Although infection is treatable with rehydration therapy, the explosive nature of outbreaks makes it difficult to treat infected patients quickly and efficiently. Effective control measures rely on prevention and preparedness. Vaccines are a necessary component in preventing cholera.

Many cholera vaccine iterations have been explored throughout the last 125 years. In the 1960s, a parenteral cellular killed cholera vaccine proved to be effective against the disease in adults, but resulted in a short protection period and caused reactogenic effects including fever and swelling [2]. Another challenge posed by a parenteral vaccine is the requirement of trained staff to use injection devices for administration [3]. Live-attenuated vaccines have also been developed, and tend to generate more robust immunological responses with fewer doses, but pose risks of reactogenic effects and necessitate determining appropriate attenuation to maintain safety while retaining antigenicity (summarized in [3]). These vaccines also require a cold chain, which is difficult to achieve in regions where electricity and other resources are limited.

An oral WCK vaccine is another option that has been studied in the effort to combat cholera. Currently, three such vaccines have been approved for human use. However, these oral WCK vaccines (Dukoral, Shanchol, and Euvichol) offer incomplete protection for a limited time in adults, and are less effective in children [3, 4]. These vaccines require two doses, two weeks apart, with a booster every two years. For Dukoral, protective efficacy is 85% for up to 6 months and then rapidly declines [5]. For Shanchol, protection is approximately 67% for 2 years and lowers to 50% over 3–5 years [5, 6]. Euvichol is the newest cholera vaccine to become prequalified by the World Health Organization and is comparable in protection to Shanchol [4]. Additionally, children under 5 years do not mount strong clinical protection from these vaccines and none of the vaccines are approved for use in children less than one year old [4, 6–8]. These vaccines elicit an immune response primarily due to the presence of lipopolysaccharide (LPS), a surface-exposed carbohydrate-based endotoxin. Young children, in general, mount a less vigorous immune response to carbohydrate immunogens than to proteins [3]. Therefore, alternative vaccine preparations are needed.

Here we report engineered *V*. *cholerae* strains that express the toxin-coregulated pilus (TCP), which is absent in currently licensed oral vaccine formulations, under the control of an inducible promoter. A preparation that includes TCP, a colonization factor required for infection [9, 10], would provide an immunogenic protein that has been demonstrated to be a protective antigen [11–17]. Our putative vaccine strains consist of two *V*. *cholerae* O1 El Tor variant biotype clinical isolates, one of the Ogawa and one of the Inaba serotype [18]. These strains have been engineered with a rhamnose-inducible promoter controlling expression of the *tcp* operon such that *tcp* gene expression can be uncoupled from the complex regulatory cascade that controls its expression in wild-type strains. Our characterization of these strains indicates their potential to help develop a new, possibly more effective WCK vaccine.

The adjustments made to the clinical isolate strains will allow for simplified preparation of the WCK cholera vaccine, and more importantly, will permit the production and inclusion of TCP in the vaccine, potentially enhancing it and improving efficacy. Particularly, we speculate that the inclusion of TCP in a WCK cholera vaccine could offer more complete protection in children.

## Materials and methods

### Bacterial strains and growth conditions

All strains and plasmids used in this study are described in Table 1.

**Table 1:** Strains or plasmids used in this study.

Strain or plasmid	Description	Reference or source
***V*. *cholerae* strains**		
O395	Classical, Ogawa, Str^R^	Laboratory Collection [19]
CL101	O395 CTX-Kmφ; Str^R^/Km^R^	Laboratory Collection [20]
O395Δ*tcpA*	O395Δ*tcpA*, Classical, Str^R^	Laboratory Collection [20]
N16961	El Tor, Inaba, Str^R^	Laboratory Collection [21]
C6706str2	C6706, spontaneous Str^R^	Laboratory Collection [10]
RM3	C6706str2, Δ15kb*ctx*	Laboratory Collection
Bgd1	El Tor Variant 01, Ogawa	Laboratory Collection [18]
Bgd5	El Tor Variant 05, Inaba	Laboratory Collection [18]
CAH170	Bgd5, Δ15kb*ctx*	This study
CAH173	Bgd1, Δ15kb*ctx*	This study
CAH182	CAH173, P_rha_*tcp*	This study
CAH184	CAH170, P_rha_*tcp*	This study
***E*. *coli* strains**		
BL21	F- ompT hsdS (rB-, mB) gal	Laboratory Collection [22]
S17-1λ*pir*	*recA*, *thi*, *pro*, *hsdR-M+* [RP4-2-Tc::Mu::Km^R^ Tn*7*] (λ*pir*); Tp^R^ Str^R^	Laboratory Collection [23]
CC118λpir	*araD139Δ(ara leu)*7697 *ΔlacX74Δpho*A20 *galK thi rpsE rpoB argE*(Am) *recA*1 (*λpir*)	Laboratory Collection [24]
**Plasmids**		
pRE118	*oriT oriV sacB aphA*, Km^R^	Laboratory Collection [25]
pCHG041	pRE118, 1035bp of Bgd1 to make Δ15kb*ctx* in Bgd1, Bgd5	This study
pCHG042	pRE118, 1025bp of tc*pH*, *tcpA* of CAH173 to make P_rha_*tcp*	This study
pCHG043	pRE118, 1025bp of tc*pH*, *tcpA* of CAH170 to make P_rha_*tcp*	This study
pCHG046	pCHG042, 2093bp of RhaRSB of BL21 to make P_rha_*tcp*	This study
pCHG047	pCHG043, 2093bp of RhaRSB of BL21 to make P_rha_*tcp*	This study

Abbreviations: Str^R^—streptomycin resistance; Km^R^—kanamycin resistance; Tp^R^—trimethoprim resistance.

All strains were maintained at -80°C in lysogeny broth (LB) containing 20% glycerol (vol/vol). Unless stated otherwise, *V*. *cholerae* strains grown under TCP-expressing conditions were grown as follows: laboratory reference control O1 classical strains (O395 and O395Δ*tcpA*) were grown in LB, starting pH of 6.5, with aeration at 30°C for 12–16 h [19, 26]; El Tor and clinical isolate strains (C6706str2, RM3, N6961, Bgd1, and Bgd5) were grown in AKI-inducing conditions as previously described [27]; and vaccine strains (CAH182 and CAH184) were grown in soy LB (traditional LB broth amended to replace tryptone with papain-digested soybean meal (Spectrum Chemical Mfg. Corp., New Brunswick, NJ) to avoid prion risk from animal material) with or without the addition of 0.1% rhamnose (vol/vol), as indicated, at 37°C for 12–16 h.

When appropriate, strains were grown with antibiotics at the following final concentrations: kanamycin 22.5 μg/ml or 45 μg/ml, polymixin B 25 μg/ml or 50 μg/ml, or with 20% sucrose (vol/vol).

### Plasmid and strain construction

Plasmids used in this study are listed in Table 1. Primers used for plasmid and strain construction are listed in S1 Table. To make cholera toxin deletions in clinical *V*. *cholerae* strains Bgd1 and Bgd5, pCHG041 was derived using the sucrose counter-selection plasmid pRE118 (ATCC^®^ 87693^™^). Primers pairs RMF1/RMR1 and RMF2/RMR2 were used to amplify Bgd1/Bgd5 chromosomal DNA outside of the *ctx* locus and the PCR amplified regions were then cloned into pRE118. Using conventional allelic exchange techniques [10], the resulting *ctx* deletion constructs (CAH170 and CAH173) contained a 15 kb deletion of the *ctx* locus, which eliminated the entire CTX genetic element (including *ctxA* and *ctxB*) and surrounding CTX-φ recognition sequences (from VC1451 to VC1475). pCHG041, CAH173 (derivative of Bgd1), and CAH170 (derivative of Bgd5), were verified by DNA sequencing. Strain RM3, a derivative of C6706str2, was produced in a similar manner (unpublished data).

To make the rhamnose-inducible *tcp* operon in the resulting “vaccine strains” (CAH182 and CAH184), plasmids were constructed in a step-wise manner. First, primers PEA002/026 were used to amplify a portion of *tcpH* upstream of the *tcpA* promoter in CAH173 and CAH170, and PEA059/060 were used to amplify a portion of *tcpA* downstream from the promoter in these strains. The amplified regions were cloned into pRE118 (ATCC^®^ 87693^™^) to produce pCHG042 and pCHG043, respectively, and plasmids were verified via DNA sequencing. Next, primers PEA027/028 were used to amplify the rhamnose promoter P_rha_ from *Escherichia coli* BL21 chromosomal DNA. The PCR amplified region was then cloned into pCHG042 and pCHG043 to produce pCHG046 and pCHG047, respectively, and these plasmids were verified via DNA sequencing. The rhamnose-inducible promoter was incorporated into CAH173 and CAH170 via allelic exchange to replace the native *tcp* promoter and produce strains CAH182 (parent strain Bgd1) and CAH184 (parent strain Bgd5) using conventional techniques [10]. Strains were verified with DNA sequencing. All DNA manipulations were performed using standard molecular and genetic techniques [28].

### Western immunoblotting and antisera

Whole-cell extracts (WCE) were assayed for total protein concentrations using a bicinchoninic acid protein assay kit (ThermoFisher, Waltham, MA). Equal amounts of total protein for each sample were resuspended in 2X sodium dodecyl sulfate-polyacrylamide gel electrophoresis buffer and samples were boiled for 10 min prior to being loaded on 16% Tris-glycine polyacrylamide gel (Invitrogen, Carlsbad, CA). Proteins were transferred to a nitrocellulose membrane via an iBlot dry blotting system (Invitrogen, Carlsbad, CA). The membrane was blocked with 3% bovine serum albumin in 1X Tris-buffered saline with 0.1% Tween (TBST). Primary antisera used included rabbit polyclonal antisera raised against TcpA [14], goat polyclonal for CtxB (Millipore, Billerica, MA), mouse monoclonal antisera for Ogawa LPS (S-20-4) [29] and for both Ogawa and Inaba LPS (72.1) [30]. Western immunoblots were visualized using the ECL (Enhanced Chemiluminescence) detection system (GE Healthcare, Little Chalfont, Buckinghamshire, UK).

Samples of purified LPS and TcpA (from laboratory collection) were used as controls. Shanchol (Shantha Biotechnics, Andhra Pradesh, India) was provided by David Sack (Johns Hopkins, Baltimore, MD).

### Cholera toxin production assay

GM_1_ ganglioside enzyme-linked immunosorbent CT assays (ELISAs) were performed on the supernatants of cultures grown under AKI-inducing conditions or soy LB rhamnose-inducing conditions, and the total ng of CT produced per ml of culture per OD_600_ unit (ng CT ml^−1^ OD_600_^−1^) was determined as previously described [31]. Purified cholera toxin B subunit (List Biological Laboratories, Campbell, CA) was used as a standard.

### Transmission electron microscopy (TEM)

Strains were grown under TCP-expressing conditions. A Formvar-coated copper grid (Electron Microscopy Supplies, Hatfield, PA) was inverted and suspended on top of a 50 μl drop from an overnight culture or liquid preparation on Parafilm for 10 min. Grids were wicked dry with Whatman filter paper, negatively stained with 0.5% phosphotungstic acid (pH 6.5) for 2 min, and wicked dry again. Stained grids were stored in a desiccated chamber until viewing. Grids were viewed using a JEOL 100CX transmission electron microscope at 100kV at magnifications up to 25,000X.

### CTX-Kmφ transduction assay

Strains were grown under appropriate TCP-expressing conditions. The CTX-Kmφ transduction assay was performed as previously described [32]. Briefly, equal volumes of CTX-Kmφ-containing supernatants and bacterial cultures were mixed and incubated in a water-bath at 37°C for 45 min. Dilutions of each sample were plated on LB agar containing kanamycin. Additionally, dilutions of bacterial cultures were plated to determine the number of input bacteria. Transduction frequency was reported as the ratio of Km^R^ test strain transductants to the number of input CFUs divided by the ratio of Km^R^ wild-type N16961 transductants to the number of input CFUs. All strains were tested in three independent experiments and data are reported as means with standard error bars.

### Heat-killing and acid treatment of vaccine strains

To heat-kill vaccine strains, CAH182 and CAH184 were grown overnight in TCP-inducing conditions with rhamnose. 1 mL aliquots of culture were centrifuged and pellets were resuspended in 200 μL 1X PBS. Microcentrifuge tubes were incubated in a dry block heater for up to 120 min at 56°C. For acid treatment of vaccine strains, again CAH182 and CAH184 were grown in inducing conditions overnight, aliquoted, and centrifuged as described above. Pellets were resuspended in 200 μL 1X PBS pH 2.0 and incubated in a water bath at 37°C for up to 120 min.

## Results

### Current oral cholera vaccine formulations do not contain TCP

**Fig 1:**
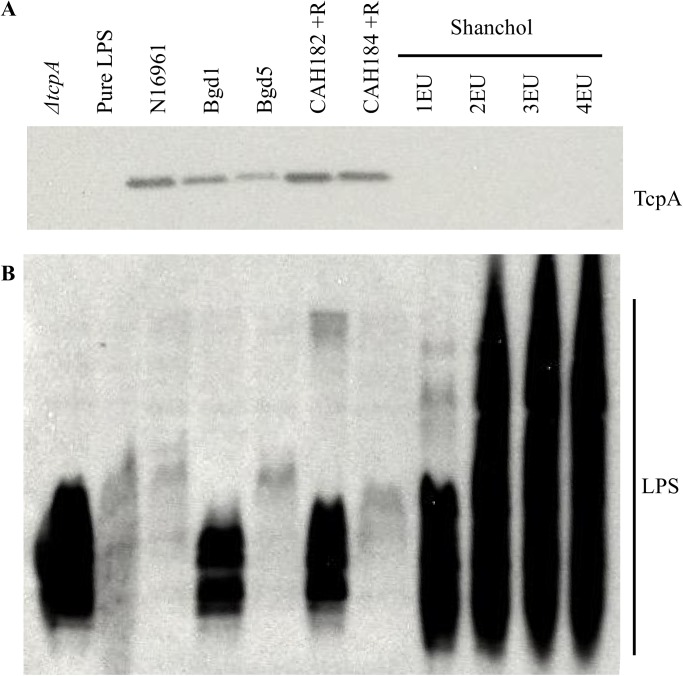
The Shanchol vaccine does not contain TcpA protein. *A*, Western immunoblot of TcpA protein present in Δ*tcpA*, wild-type N16961 (El Tor), clinical El Tor variant strains Bgd1 (Ogawa) and Bgd5 (Inaba), and rhamnose-inducible *tcp* strains in the Bgd1 and Bgd5 background with cholera toxin genes deleted (referred to as CAH182 and CAH184, respectively; +R, with 0.1% rhamnose) as compared to the Shanchol vaccine in ELISA units (EU). *B*, Western immunoblot of LPS present in these strains compared to Shanchol, probed with anti-LPS antiserum 72.1 (detects Ogawa and Inaba LPS).

The toxin-coregulated pilus (TCP) is a filamentous surface component of *V*. *cholerae* that is produced in significant quantities only under certain environmental conditions. While TCP can be produced under specific laboratory cell growth conditions [19, 26, 27], the way in which the *V*. *cholerae* strains contained in the oral, whole cell vaccine Shanchol are grown does not result in production of TCP, as evidenced by a lack of the TCP major pilin protein TcpA on a western immunoblot (Fig 1A, right side). It is thought that the primary immune response to this vaccine is likely due to the abundance of the outer membrane carbohydrate complex lipopolysaccharide (LPS) (Fig 1B).

### Clinical variant strain selection for enhanced WCK cholera vaccine

**Fig 2:**
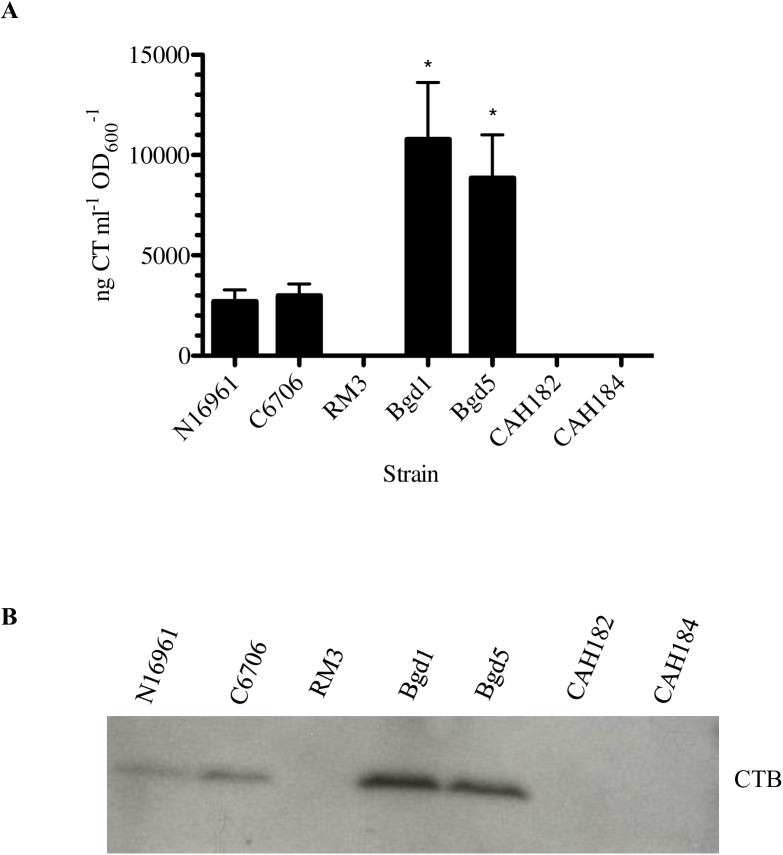
Vaccine strains do not produce cholera toxin. *A*, Cholera toxin production in the rhamnose-inducible *tcp* strains, and *ctx* knockout strains CAH182 and CAH184 as compared to wild-type N16961, wild-type C6706, RM3, which is the same *ctx* region deletion in the C6706 background, Bgd1 and Bgd5 strains. ngCTml^-1^OD_600_^-1^ ELISA measurements for three independent experiments presented as means with standard errors. A two-tailed standard *t* test yielded *P* values of <0.05 when CTX production of Bgd1 (*) and Bgd5 (*) were compared to all other strains. Bgd1 and Bgd5 were not significantly different from each other. *B*, Western immunoblot of cholera toxin B subunit (CTXB) in the listed strains, thereby confirming deletion of the genes encoding cholera toxin.

Two *Vibrio cholerae* O1 El Tor variant biotype clinical strains were selected as candidate strains to be included in an enhanced WCK cholera vaccine. While El Tor is the dominant biotype in current cholera incidences, classical biotype features (typically genetically classical cholera toxin genes) have emerged in the form of hybrid El Tor variant strains [18, 33–35]. These new pathogenic, clinically isolated variants have spread throughout Asian and African countries, appear to cause more severe disease and higher cases of fatalities [5], and are important to consider in future cholera vaccine development [36]. The selected strains were isolated from patients at Matlab Hospital in Bangladesh and were originally obtained from the International Center for Diarrheal Disease Research, Bangladesh (ICDDR,B) [18]. These strains, Bgd1 and Bgd5, caused severe dehydration and acute watery diarrhea in the infected patients and produce high levels of cholera toxin (Fig 2A and 2B). Bgd1 and Bgd5 are Ogawa and Inaba serotypes, respectively (confirmed via western protein immunoblot in S1 Fig), classified by variations in the O-antigenic component of LPS [37]. Both strains contain a single copy of the cholera toxin genes (*ctxA* and *ctxB*) on the large chromosome only [18].

### Modified vaccine strains do not produce cholera toxin

After selection of the clinical variant strains, we first deleted the *ctxA and ctxB* genes, plus the surrounding regions of the genome in each strain, resulting in a ~15 kb deletion of the *ctx* locus (from VC1451 to VC1475). This deletion eliminates the entire CTX genetic element and surrounding CTX-φ recognition sequences [32], such that CTX-φ cannot reincorporate the cholera toxin genes back into the chromosome in the final vaccine strains. We confirmed the absence of cholera toxin production in the final vaccine strains via GM_1_ ganglioside ELISA for cholera toxin (Fig 2A) and western immunoblot for cholera toxin B protein (Fig 2B).

### Vaccine strains contain rhamnose-inducible *tcp* and produce functional pili

Following deletion of the cholera toxin genes, we placed the *tcp* operon under the control of the *E*. *coli* rhamnose promoter by replacing the native *V*. *cholerae tcp* promoter with P_rha_ from *E*. *coli* strain BL21, thus placing expression of the *tcp* operon under control of rhamnose. This approach allows for the inclusion of the protective antigen, TCP, in the vaccine formulation simply by growing the bacteria in the presence of 0.1% rhamnose.

**Fig 3:**
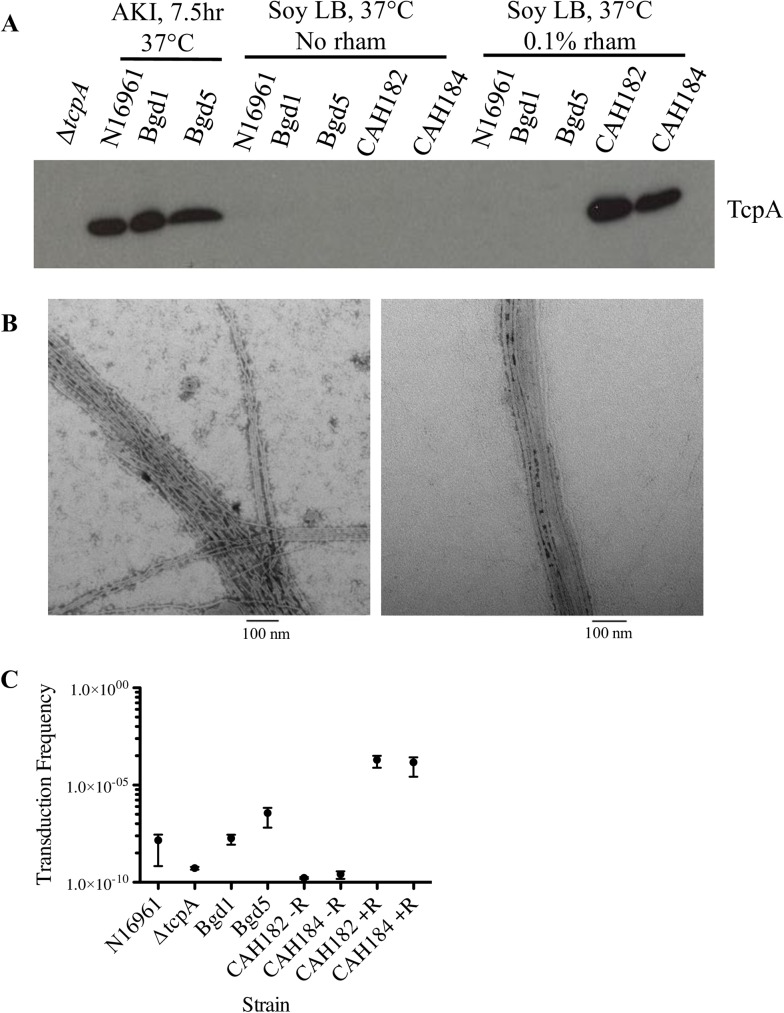
TcpA and pilus production in vaccine strains. *A*, Western immunoblot of TcpA production in rhamnose-inducible *tcp*, *ctx* knockout strains CAH182 and CAH184 as compared to wild-type N16961, Bgd1 and Bgd5 strains under AKI-inducing conditions as compared to growth in soy LB (traditional lysogeny broth amended to replace tryptone with papain-digested soybean meal to avoid prion risk from animal material) with or without addition of 0.1% rhamnose. *B*, Transmission electron microscopy of phosphotungstic acid-negatively stained pili produced by CAH182 (left) and CAH184 (right) after growth in soy LB with 0.1% rhamnose. *C*, CTX-Kmϕ phage transduction via TCP in wild-type N16961, Bgd1 and Bgd5 in AKI growth conditions and vaccine strains CAH182 and CAH184 with (+R) and without (-R) 0.1% rhamnose grown in soy LB. Data from three independent experiments +/- standard errors. A two-tailed standard *t* test yielded no significant differences among strains.

To verify production of TCP that is dependent upon rhamnose addition, the final vaccine strains CAH182 and CAH184 (parental strains are Bgd1 and Bgd5, respectively) were grown in an animal-free medium (soy-based LB broth) at 37°C overnight with or without the addition of 0.1% rhamnose (vol/vol). Whole cell extracts (WCE) were assayed via western immunoblot for the presence of TcpA, the major pilin that forms TCP, using anti-TcpA anti-serum. Both CAH182 and CAH184 produced stable TcpA protein only when grown in the presence of rhamnose (Fig 3A). Parental strains Bgd1 and Bgd5, along with control *V*. *cholerae* El Tor strain N16961, only produced TcpA when grown in AKI-inducing conditions as previously described [27] and did not produce TcpA in soy LB with or without the inclusion of rhamnose (Fig 3A).

Additionally, pili production was evident in the rhamnose-induced vaccine strain cultures when whole cell extracts were negatively stained with phosphotungstic acid (PTA) and viewed using transmission electron microscopy (TEM) (Fig 3B).

Not only are pili produced by the induced vaccine strains, they are also functional, as demonstrated via a phage transduction assay (Fig 3C) performed as previously described [32]. CAH182 and CAH184 were grown with and without 0.1% rhamnose in soy LB overnight at 37°C, while control strains were grown in AKI-inducing conditions. Fig 3C shows that the vaccine strains are susceptible to CTX-Kmφ infection at high levels when *tcp* expression is induced via rhamnose. The presence of TCP allows for uptake of the Km-resistant bacteriophage, which replicates as a plasmid [32], permitting growth of bacteria on LB agar containing kanamycin.

### TCP is produced in larger culture volumes

**Fig 4:**
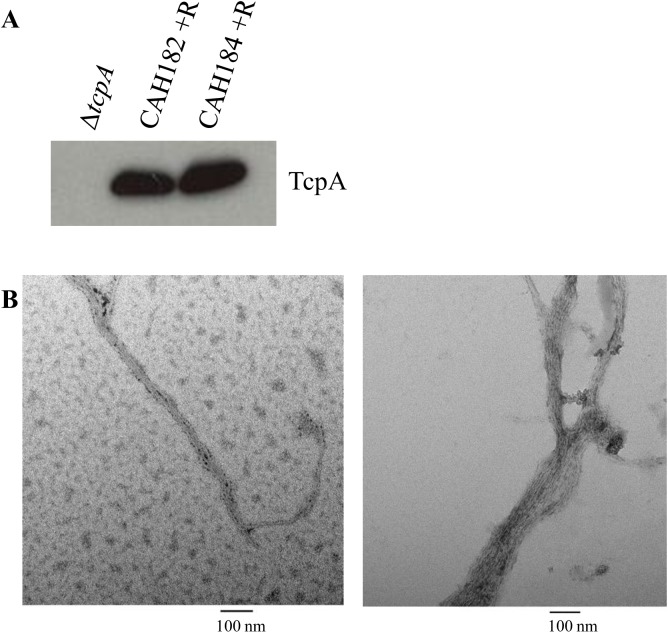
Vaccine strains produce TCP in 1L growth volumes. *A*, Western immunoblot analysis of TcpA from CAH182 and CAH184 induced with 0.1% rhamnose (+R) in soy LB grown in large (1L) volumes. *B*, Pili produced by CAH182 (left) and CAH184 (right) in the above conditions as seen with transmission electron microscopy and negative staining.

A consideration for vaccine production is expression of the antigens in large culture volume in order to mass-produce the final vaccine formulation. To ensure that the rhamnose-inducible strains CAH182 and CAH184 continue to produce TCP in larger culture volumes, we grew 1-liter overnight cultures of each bacterial strain induced with 0.1% rhamnose at 37°C with agitation. Fig 4A (western immunoblot for TcpA) and 4B (TEM images) show that the vaccine strains CAH182 and CAH184, when grown in the presence of 0.1% rhamnose, produce TCP, even if grown in larger volumes of soy LB medium at 37°C, consistent with the strains potentially being amendable for mass vaccine production.

### TCP produced by vaccine strains can withstand heat-killing and acid treatment

A possible cholera vaccine formulation would contain the strains CAH182 and CAH184 grown in *tcp*-expressing conditions, followed by heat-killing the strains at 56°C for one hour. To ensure heat-killing the bacteria does not disrupt the integrity of TCP, strains were grown in inducing conditions overnight and whole cell extracts were centrifuged and resuspended in PBS, followed by incubation of samples at 56°C for 15, 30, 60, and 120 minutes. Heat-killing was verified by plating approximately 5x10^10^ colony forming units (CFU) onto an LB agar plate and incubating for 48 hours at 37°C, followed by incubation at room temperature for an additional four days. Zero CFU were recovered after the incubation period for all time points, indicating killing of 100% of the bacteria in as little as 15 min at 56°C (data not shown).

**Fig 5:**
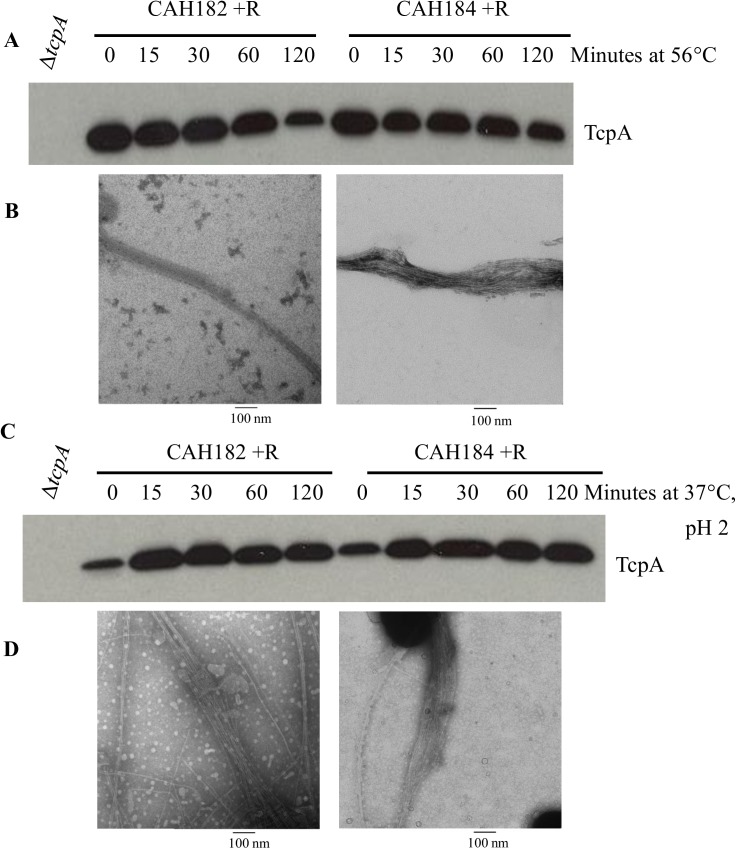
Pili produced by vaccine strains are stable after incubation in high heat or an acidic medium. *A*, Western immunoblot analysis for TcpA after heat-killing of the vaccine strains CAH182 and CAH184 at 56°C over two hours following overnight growth in soy LB with 0.1% rhamnose (+R) at 37°C. *B*, Transmission electron microscopy results of the negatively-stained pili after heat-killing for 60 minutes (CAH182, left and CAH184, right). *C*, Western immunoblot analysis of TcpA after treatment of the vaccine strains CAH182 and CAH184 with acid (pH 2.0) up to two hours at 37°C after overnight growth in soy LB at 37°C with 0.1% rhamnose (+R). *D*, Transmission electron microscopy results of negatively-stained pili after treatment with acid for 120 minutes (CAH182, left and CAH184, right).

Heat-killed samples were then analyzed via SDS-PAGE and a western blot for TcpA indicated that TcpA protein was stable for up to one hour after heat-killing (Fig 5A). Intact pili were viewed via TEM in the 60 min killed samples (Fig 5B). However, at 120 min incubation at 56°C, TcpA levels were reduced by ~50% (Fig 5A) and few pili were visible by TEM (data not shown). Regardless, 60 min at 56°C was more than sufficient to ensure killing of the bacterial strains while retaining TCP.

The integrity of TCP was also tested after incubation in an acidic environment to assess its ability to withstand the acidic gastric environment encountered upon ingestion of an oral cholera vaccine. Overnight, induced cultures of the vaccine strains were centrifuged and resuspended in PBS with a pH of 2. The samples were incubated in the acidic PBS for 15, 30, 60, and 120 minutes at 37°C. Western immunoblot analysis and TEM imaging showed that acid treatment did not disrupt the stability of TcpA nor the integrity of whole pili for all incubation periods (Fig 5C and 5D, respectively).

## Discussion

We have developed strains of *V*. *cholerae* that show potential to be used in a new oral, whole cell killed cholera vaccine that includes two clinical isolate O1 El Tor variant strains, an Ogawa and Inaba serotype, each containing cholera toxin gene deletions and a rhamnose-inducible *tcp* operon, such that a vaccine can be prepared that is non-toxigenic and includes the protective antigen toxin-coregulated pilus following culture in an animal-free medium supplemented with 0.1% rhamnose in addition to the cell wall antigens found in the current killed oral vaccines. This strategy simplifies the way in which the vaccine is prepared, as existing vaccine formulations contain some cholera toxin that needs to be removed during preparation of the formulations [3], while our vaccine strains are entirely devoid of cholera toxin. Additionally, the vaccine strains, CAH182 and CAH184, produce robust, functional pili when induced with rhamnose. This antigen is completely lacking in the currently available WCK formulations. Our strains are also potentially amenable for scaled-up vaccine production, as TCP is still produced in larger culture volumes. Further assessment of TCP production as a result of bacterial growth in larger bioreactors would reaffirm the potential for successful scale-up production, but was not performed for this study. Moreover, because the whole *tcp* operon is induced, other TCP proteins will be expressed, such as the novel and more recently discovered colonization factor, TcpF, which, like TCP, is required for infection [20, 38]. These strains also express two serotypes of LPS from the most widespread *V*. *cholerae* strains. Experiments comparing the putative TCP-enhanced vaccine strains to non-TCP expressing *V*. *cholerae* strains in an animal model would be an important follow up to this preliminary study.

The final vaccine formulation can be easily prepared by heat-killing the strains for one hour at 56°C, which we have shown does not disrupt the integrity of the pili produced in the volumes tested. This finding is unlike the current inactivated cholera vaccines, which contain both heat-killed and formalin-killed strains, and necessitate the removal of the formalin used in production [3]. We have also shown in this study that exposure to an acidic environment, reminiscent of the gastric environment that would be encountered by an oral vaccine, does not disrupt the integrity of the pili produced in the vaccine. This observation indicates that the TCP would remain intact during passage through the stomach.

Our putative vaccine strains should result in increased efficacy due to the presence of TCP, especially, we hope, in children that do not mount an effective immune response against carbohydrate antigens, which represent the protective antigen (LPS) component in the current vaccines [3]. Although children do not mount a good immune response to carbohydrates, they do to proteins like TCP, which suggests a WCK vaccine comprised of our *V*. cholerae strains could prove more effective for populations under 5 years of age compared to current formulations. Furthermore, an enhanced WCK vaccine containing additional protective antigens could lead to higher protection and efficacy in adults as well.

## Supporting information

S1 Fig Western immunoblot analysis of LPS of wild-type O395, wild-type N16961, Bgd1 and Bgd5, and the vaccine strains CAH182 and CAH184, confirming Ogawa and Inaba serotypes. LPS-antibody S-20-4 reacts with Ogawa serotype only (left), while the 72.1 antibody binds both Ogawa and Inaba serotypes (right).
(DOCX)

S1 Table Primers used in study. (DOCX)
