# Supplementary figures and images for: Production of putative enhanced oral cholera vaccine strains that express toxin-coregulated pilus
Source: PLoS One. 2017 Apr 6;12(4):e0175170. doi: 10.1371/journal.pone.0175170 (PMC5383245; doi:10.1371/journal.pone.0175170)

**S1 Fig.**


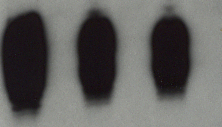


Ogawa LPS only

(S-20-4)

O395

N16961

Bgd1

Bgd5

CAH182

CAH184

LPS


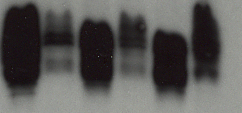


O395

N16961

Bgd1

Bgd5

CAH182

CAH184

Ogawa and Inaba LPS

(72.1)

Supplement: S1 Fig — LPS-antibody S-20-4 reacts with Ogawa serotype only (left), while the 72.1 antibody binds both Ogawa and Inaba serotypes (right). (DOCX) [file pone.0175170.s001.docx]
