# Supplementary material for: Production of putative enhanced oral cholera vaccine strains that express toxin-coregulated pilus
Source: PLoS One. 2017 Apr 6;12(4):e0175170. doi: 10.1371/journal.pone.0175170 (PMC5383245; doi:10.1371/journal.pone.0175170)
Supplement: S1 Table — (DOCX) [file pone.0175170.s002.docx]

**S1 Table**

| **Name** | **Nucleotide sequence (5**’ **to 3’)** |
| --- | --- |
| RMF1 | GATCGGAATTCTATGGTTCATATCGGTGACGG |
| RMR1 | TGACGAAGCTTATCAGCGCG |
| RMR2 | GATCGAAGCTTCCTGTCAATACCCATTAGTG |
| RMR2 | GATCGTCTAGATCGGTGATGTCTTTGCTCAG |
| PEA002 | GATCGGAGCTCGGTAATCTATCCAATGTTT |
| PEA026 | GATCGGCGGCCGCACTCAAATATTTCCGGAGTGGAGATCG |
| PEA027 | GATCGGCGGCCGCTGAGCCTCGGTTGTGTGGTG |
| PEA028 | GATCGGGTACCACACGAATTTCATTACGACCAGTC |
| PEA059 | GATCGGCGGCCGCAAACCCGGTACCAGTGCGTATTGCTTTC |
| PEA060 | GATCGTCTAGAAAGCCACCGACTGTAATTGCGAATGC |
